# Perceptual Advantage of Animal Facial Attractiveness: Evidence From b-CFS and Binocular Rivalry

**DOI:** 10.3389/fpsyg.2020.01670

**Published:** 2020-07-10

**Authors:** Junchen Shang, Zhihui Liu, Hong Yang, Chengyu Wang, Lingya Zheng, Wenfeng Chen, Chang Hong Liu

**Affiliations:** ^1^College of Psychology, Liaoning Normal University, Dalian, China; ^2^Department of Psychology, Renmin University of China, Beijing, China; ^3^Department of Psychology, Bournemouth University, Poole, United Kingdom

**Keywords:** facial attractiveness, binocular rivalry, breaking continuous flash suppression, preconscious processing, by-products hypothesis

## Abstract

Research has shown that attractive human faces enjoy an advantage in both conscious and preconscious processing. Here we examined whether this preference for attractiveness is exclusive to human faces by measuring participants’ sensitivity to the attractiveness of cat and tiger faces. Experiment 1 measured the time taken to break continuous flash suppression (b-CFS), whereas Experiment 2 measured the dominant time in binocular rivalry (BR). The results showed that attractive cat faces were detected more quickly (Experiment 1) and dominated for longer time in visual awareness (Experiment 2). However, no effect of attractiveness was found for tiger faces in Experiment 1, while attractive tiger faces also dominated for longer time in visual awareness in Experiment 2. The results provide first evidence that the preference for attractive animal faces can be shown involuntarily or without apparent conscious control. The findings suggest that human preference for facial attractiveness may contain an aesthetic element rather than being a purely adaptive means for mate choice.

## Introduction

Research has shown that humans’ fascination with facial beauty may have a strong biological basis, and the preference for attractive faces is already present at birth. Infants as young as 3-day-olds to 6-month-olds look longer at attractive faces that are considered as being attractive by adults (Samuels and Ewy, 1985; Langlois et al., 1987; Slater et al., 1998; Rubenstein et al., 1999), showing the same preference as adults (Kranz and Ishai, 2006; Lindell and Lindell, 2014; Leder et al., 2016). The early onset demonstrates a hardwired interest in attractive faces, as cultural environment should have a minimal influence at this stage. Currently there are two contrasting accounts for the preference: one sees it as an adaptive mechanism for mate choice (Little et al., 2011; Lindell and Lindell, 2014; Foo et al., 2017b) whereas the other treats it as a by-product of how brains process information (see Rhodes, 2006, for a review).

From the evolutionary perspective, facial beauty is an honest maker of genetic quality, such as health and resistance to diseases, which implies an important role in mate choice. Bilateral symmetry, for example, as one of the contributing factors in facial attractiveness, appears to be associated with health and developmental stability (Zaidel et al., 2005; Fink et al., 2006; Foo et al., 2017a).

The biological significance of facial beauty may also explain why attractive faces tend to attract greater attention (Sui and Liu, 2009; van Hooff et al., 2011; Chen et al., 2012; Nakamura and Kawabata, 2014). Evidence from imaging studies show that attractive faces activate orbitofrontal cortex and the nucleus accumbens regions of the brain that are known to be associated with rewards and pleasure (Aharon et al., 2001; O’Doherty et al., 2003; Bray and O’Doherty, 2007; Winston et al., 2007; Tsukiura and Cabeza, 2011; Hahn and Perrett, 2014). Facial attractiveness can be appraised even when a face image is presented for merely 13 ms using forward and backward visual masking (Olson and Marshuetz, 2005). There is also evidence that appraisal of facial attractiveness can occur with little voluntary control. Mo et al. (2016) used a binocular rivalry (BR) paradigm to investigate the preconscious processing of facial attractiveness. Participants were simultaneously shown a face to one eye but a house to the other. Results showed that attractive faces persisted in the percept longer than unattractive faces in the BR task, suggesting that attractive faces are more likely to be maintained in conscious vision even though the observer typically has little voluntary control over how long each of the two visual inputs will enter the conscious visual experience in the process. The longer experience for attractive faces despite the involuntary control in BR task may suggest an automatic modulation of the two percepts without experiencing a conscious effort. In a similar fashion, recent studies using a breaking continuous flash suppression (b-CFS) paradigm have also demonstrated that attractive faces broke through continuous flash suppression more quickly than unattractive faces (Hung et al., 2016; Nakamura and Kawabata, 2018). Again, authors from these studies argue that their results provide evidence for preconscious appraisal of the facial attractiveness. Some authors (Moors et al., 2017, 2019) dispute whether the b-CFS paradigm can truly reveal unconscious processing.

However, the pre-attentive appraisal of facial beauty is not by itself a proof of its evolutionary significance. Indeed, an alternative to the mate quality account is the idea that the sensitivity to facial beauty is merely a by-product of the natural selection, which may have little to do with mate choice (Rhodes, 2006; Quinn et al., 2008). Consistent with this idea, factors that are important in the evaluation of facial beauty are often also essential to the attractiveness of non-face stimuli. For example, averageness is not only linked to attractiveness of human faces, but also to attractiveness of fish and birds (Halberstadt and Rhodes, 2003). Moreover, Quinn et al. (2008) found that 3- to 4-month-olds’ looking time on attractive cat and tiger faces was significantly longer than less attractive cat and tiger faces, suggesting that infants’ preference for beauty is not limited to human faces. These findings are not easily explained by the mate choice account.

However, some may argue that although human faces are not the only objects for attractiveness appraisal, human observers may be more fluent in processing facial attractiveness. For example, Olson and Marshuetz (2005) have suggested that perhaps only facial attractiveness is appraised quickly and preconsciously, whereas judgments for other types of attractive stimuli may be slower. For other types of attractive stimuli, they used Halberstadt and Rhodes (2003) animal stimuli as examples. Thus, although preference for facial attractiveness seems to be generalized to other species, it remains unclear whether preference for animal attractiveness displays a same level of sensitivity as that found in appraisal of human facial attractiveness. It is not known, in particular, whether what was demonstrated in preconscious processing of human facial attractiveness can also be demonstrated with animal faces. If non-human attractiveness is perceived with the same level of sensitivity, it would further call into doubt the idea that preference for human facial attractiveness is exclusively linked to the function of mate choice.

The first objective of this study was to examine this question. Also, because Quinn et al. (2008) have only studied preference for cat and tiger faces in infants, our study of adult perception of facial attractiveness in these animals should reveal whether a similar pattern of preference for faces of cats and tigers maintained after subsequent years of development. Children’s face perception is known to develop through perceptual narrowing (Nelson, 2001). For example, infants of 6-month olds are able to discriminate among monkey faces as well as human faces, but this ability is lost after 9 months of age due to the narrowing of experience tuned to human faces (Pascalis et al., 2002). It is unclear whether adults would show equal preference for attractive cat and tiger faces as infants because children typically grow up with the far more frequent contacts with cats than with tigers. The tendency to perceive cuteness in cats may be further shaped by the popular pet culture, which should also contribute adults’ perception. In contrast, tigers are less accessible and are typically seen only on television, or in zoos and safaris. These factors may influence processing of facial attractiveness in these animals. With these questions in mind, the present study examined the effect of attractiveness in cat and tiger faces using the b-CFS (Experiment 1) and the BR (Experiment 2) paradigms.

## Experiment 1

### Materials and Methods

This experiment employed the b-CFS paradigm (Tan and Yeh, 2015) to test whether the suppression time of animal faces varies with levels of facial attractiveness. Suppression time is defined as the duration for the face image to emerge from a continuous suppression noise (Jiang et al., 2007). The noise patterns were presented to the participant’s dominant eye, while the face was presented to the non-dominant eye, with contrast increasing gradually from 0 to 100%. Participants had to report whether the face was presented as soon as they detected the target. Then they had to report whether the face was on the left or the right side of the screen.

#### Participants

Forty-six participants (*M*_age_ = 20, *SD*_age_ = 1.82) were recruited for the experiment. All reported normal or corrected-to normal vision and were naïve to the purpose of the study. They were reimbursed 30 RMB for their participation. Both experiments in this study were approved by the Institutional Review Board of Liaoning Normal University, China. Informed consent was obtained from each participant prior to the experiments. Due to a program error, data from seven participants were not recorded. In addition, the data from two participants whose accuracy results were three standard deviations below the group mean were also excluded. The remaining 37 participants (33 female) were included in the final analysis.

#### Apparatus and Stimuli

For the b-CFS task, instructions and stimuli were presented on a 17-inch Lenovo CRT monitor with a refresh rate of 85 Hz, and a screen resolution of 1024 × 768 pixels. Stimuli were controlled by E-Prime Version 2 on a HP 280 Pro G2 MT. A chin rest was used to maintain the participant’s head position. Stimuli were shown through a mirror stereoscope (provided by Beijing Fistar Technology Co., Ltd.). The 10 colorful Mondrians stimuli generated by MATLAB were adopted from Jiang et al. (2007), which were used widely in several other studies (Jiang et al., 2009; Zhou et al., 2010; Liu et al., 2016). The size is 130 × 130 pixels with a dimension of 4.1 × 4.1°. They changed every 100 ms (10 Hz) as suppressor through the mirror stereoscope. A frame (11.8° × 11.8°) that extended beyond the outer border of the stimulus and fixation cross (1.1° × 1.1°) was presented to facilitate stable fusion of the two images. For the rating task, instructions and stimuli were presented on a 19-inch Lenovo LCD monitor with a resolution of 1440 × 900 pixels and a refresh rate of 60 Hz.

The 72 cat and the 72 tiger images were collected from the Internet. Images were cropped to only include the face and ears. All images were converted to grayscale, and were presented on a neutral gray background. All were also normalized to have the same mean luminance and contrast using Adobe Photoshop CS color match tool (please see Anderson et al., 2011 for this method).

**FIGURE 1:**
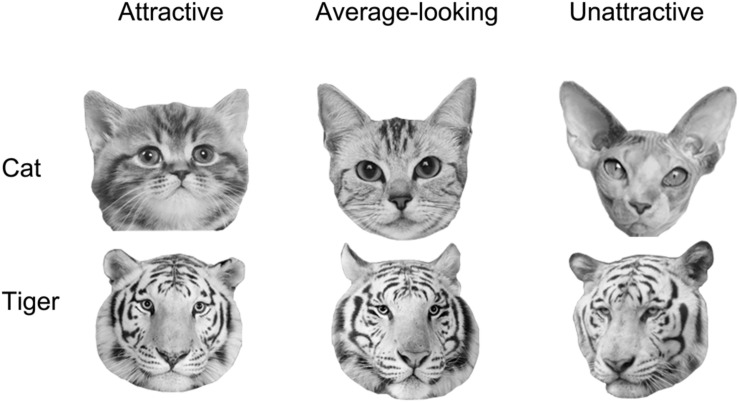
Examples of the cat and tiger face stimuli used in the experiments.

We classified cat and tiger faces into three levels of attractiveness (attractive, average-looking, and unattractive) according to the ranking order of their mean ratings obtained from this experiment. The attractiveness was defined on the mean ratings across all participants in the attractiveness-rating task. The mean ratings for these are given in first row of Table 1. Figure 1 shows some example faces. The top 24 and the bottom 25 cat faces were classified as being attractive and unattractive, respectively. The remaining 23 were classified as average-looking cat faces. The tiger faces were classified using the same method, which created 23 attractive, 25 average-looking, and 24 unattractive tiger faces. There was no overlap between ratings for each of the categories for either cat or tiger faces.

**TABLE 1:** Mean attractiveness ratings of cat and tiger faces in Experiments 1 and 2 based on a 7-point scale, ranging from 1 (least attractive) to 7 (most attractive).

	Cat faces			Tiger faces		
	Attractive	Average-looking	Unattractive	Attractive	Average-looking	Unattractive
Experiment 1	5.33 (0.44)	3.56 (0.44)	2.03 (0.34)	4.49 (0.26)	3.65 (0.20)	2.64 (0.34)
Experiment 2	5.68 (0.27)	3.82 (0.26)	1.71 (0.20)	4.69 (0.17)	3.52 (0.19)	2.32 (0.21)

*Standard deviations are given in parentheses.*

#### Design

We employed a within-subject design. The independent variables were Face Type (cat vs. tiger) and Attractiveness (attractive, average-looking, unattractive). The dependent variable was the mean suppression time.

#### Procedure

Participants were tested individually. They first completed the b-CFS task and then followed by the rating task. The viewing distance in both tasks was 57 cm.

##### The b-CFS task

**FIGURE 2:**
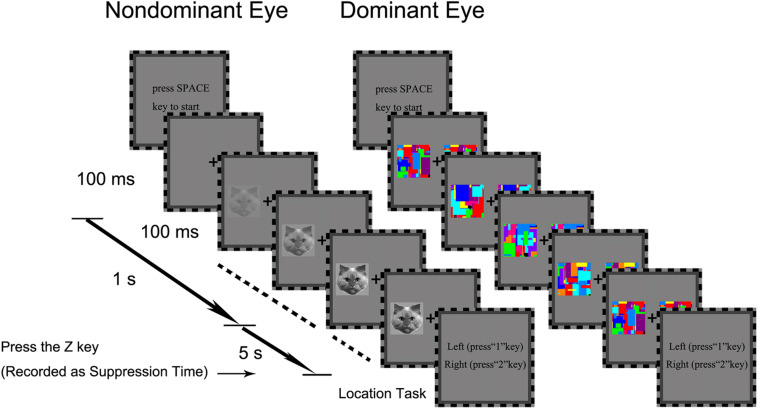
The trial procedure of the b-CFS task in Experiment 1. The contrast of a target image (e.g., the cat face in this example) presented to the non-dominant eye was gradually increased to compete with a dynamic noise pattern (Mondrians) presented to the dominant eye. The contrast of the target image was linearly ramped up from 0 to 100% within a period of 1 s starting from the beginning of the trial, and then remained constant until the observer made a response to indicate the side on which the target appeared. The trial ended if the participant did not response within 6 s from the beginning for the trial.

We first established eye dominance for each participant using the hole-in-the-card test (Dolman method; Anderson et al., 2012). Each trial began with a press on the SPACE key. A central fixation cross was presented to both eyes. Simultaneously, two colorful Mondrians were presented to the participant’s dominant eye. As illustrated in the middle panel of Figure 2, the two Mondrians were presented simultaneously to the left and right of the fixation cross at full contrast, and continued to flash by continuously alternating with new Mondrians at 10 Hz till the end of the trial. Meanwhile, a 2.1° × 2.5° target image was presented to the non-dominant eye either to the left or right side of the fixation cross, within the region corresponding to the location of the Mondrians. The contrast of this target image increased gradually from 0 to 100% at rate of 10% per 100 ms within a 1-s frame. The target remained on the screen at full contrast after the initial 1 s period until participants pressed the Z key to stop when the target was detected. After this, they were required to press “1” or “2” key to report whether the target was presented on the left or right side of the fixation cross after the detection task. The trial ended if the participants did not respond after 6 s.

The b-CFS task consisted of two blocks: one for the cat faces, and the other for the tiger faces. The order of the blocks was counterbalanced between participants. Each block had 144 trials. A rest was given after each block. The target was presented to the left or right location equally often within a block. All trials were presented in a random order. Prior to the experimental trials, participants were given 24 practice trials at the beginning of each block with example stimuli that were not used in the subsequent experimental trials.

##### The attractiveness-rating task

Following the b-CFS task, participants were asked to rate the attractiveness of the 144 faces used in the b-CFS task on a 7-point scale from 1 (not attractive at all) to 7 (very attractive). On each trial, a face was presented in the center of the screen until the response had been made. Participants completed two blocks, one with cat faces and the other with tiger faces. The faces within each block were presented in a random order. The order of two blocks was counterbalanced across participants.

##### The online-rating task for pleasure and arousal

Since emotional information has been shown to influence unconscious processing of stimuli (Morris et al., 1998; Dimberg et al., 2000; de Gelder et al., 2005; Yang et al., 2007; Adams et al., 2010), it is important to rule out this possibility in order to claim that the attractiveness of suppressed animal faces indeed played a role in gating suppression time. 29 subjects (4 male, *M*_age_ = 24.48, *SD*_age_ = 1.88) were recruited to rate the pleasure and arousal of the all 144 faces by a 9-point Self-Assessment Manikin (SAM; Bradley and Lang, 1994; Morris, 1995) scale. The faces and SAM were presented online by wjx.cn^1^. A face and a SAM scale appeared on the screen at the same time. The participants needed to evaluate pleasure and arousal based on their gut feeling about the face. Regarding the pleasure scale, 1 indicated that the face made the participant feel the most unpleasant, 5 meant neutral, and 9 meant the face made the participant feel the most pleasant. Regarding the arousal scale, 1 indicated that the face made the participant feel the least awakened, excited, and tense, 5 meant neutral, and 9 meant that the face made the participant feel the most awakened, excited, and tense. The mean ratings for pleasure and arousal are given in Tables 2, 3.

**TABLE 2:** Mean pleasure ratings of cat and tiger faces in Experiments 1 and 2 based on a 9-point SAM scale, ranging from 1 (the most unpleasant) to 9 (the most pleasant).

	Cat faces			Tiger faces		
	Attractive	Average-looking	Unattractive	Attractive	Average-looking	Unattractive
Experiment 1	6.39 (0.53)	4.35 (0.59)	2.84 (0.56)	5.17 (0.67)	4.51 (0.52)	3.74 (0.81)
Experiment 2	6.81 (0.49)	4.65 (0.38)	2.54 (0.58)	5.66 (0.60)	4.76 (0.63)	3.20 (0.56)

*Standard deviations are given in parentheses.*

**TABLE 3:** Mean arousal ratings of cat and tiger faces in Experiments 1 and 2 based on a 9-point SAM scale, ranging from 1 (the least awakened, excited, and tense) to 9 (the most awakened, excited, and tense).

	Cat faces			Tiger faces		
	Attractive	Average-looking	Unattractive	Attractive	Average-looking	Unattractive
Experiment 1	6.10 (0.41)	5.02 (0.39)	5.19 (0.47)	5.57 (0.45)	5.19 (0.43)	4.95 (0.71)
Experiment 2	6.47 (0.32)	5.06 (0.31)	5.07 (0.46)	5.81 (0.39)	5.28 (0.44)	5.16 (0.82)

*Standard deviations are given in parentheses.*

#### Results and Discussion

##### Analysis of inter-rater reliability

Kendall’s W was calculated to establish a measure of inter-rater reliability. For attractiveness ratings: for all pictures, Kendall’s W was 0.41, χ^2^ = 2159.31, *df* = 143, *p* < 0.001. When the two face categories were calculated separately, Kendall’s W was 0.56 for cats, χ^2^ = 1474.13, *df* = 71, *p* < 0.001, and 0.23 for tigers, χ^2^ = 610.85, *df* = 71, *p* < 0.001. For pleasure ratings: for all pictures, Kendall’s W was 0.38, χ^2^ = 1589.62, *df* = 143, *p* < 0.001. When the two face categories were calculated separately, Kendall’s W was 0.49 for cats, χ^2^ = 1013.02, *df* = 71, *p* < 0.001, and 0.27 for tigers, χ^2^ = 556.44, *df* = 71, *p* < 0.001. For arousal ratings: for all pictures, Kendall’s W was 0.11, χ^2^ = 452.64, *df* = 143, *p* < 0.001. When the two face categories were calculated separately, Kendall’s W was 0.09 for cats, χ^2^ = 189.08, *df* = 71, *p* < 0.001, and 0.13 for tigers, χ^2^ = 259.95, *df* = 71, *p* < 0.001.

Three two-way analysis of variance (ANOVA) were conducted on the mean ratings of attractiveness, pleasure and arousal respectively, using Attractiveness and Face Type as independent variables.

##### Analysis of attractiveness ratings

The main effect was significant for Attractiveness, *F*(2, 138) = 664.98, *p* < 0.001, η*_*p*_*^2^ = 0.91, but not for Face Type, *F*(1, 138) = 0.79, *p* = 0.377, η*_*p*_*^2^ = 0.01. These were qualified by a significant interaction, *F*(2, 138) = 53.35, *p* < 0.001, η*_*p*_*^2^ = 0.44. Analysis of simple effect showed that the attractive cat faces were rated as more attractive than the attractive tiger faces, *F*(1, 45) = 63.26, *p* < 0.001, η*_*p*_*^2^ = 0.58, and the unattractive cat faces were rated as more unattractive than the unattractive tiger faces, *F*(1, 47) = 38.72, *p* < 0.001, η*_*p*_*^2^ = 0.45, but the average-looking cat and tiger faces were not different from one another, *F*(1, 46) = 0.72, *p* = 0.401, η*_*p*_*^2^ = 0.02. Simple effects analyses were also carried out for cat and tiger faces separately. The effect of attractiveness was significant for cat faces, *F*(2, 69) = 403.91, *p* < 0.001, η*_*p*_*^2^ = 0.92. Pairwise comparison (Bonferroni corrected) showed that the attractive cat faces were rated as more attractive than the average-looking cat faces (*p* < 0.001), which was in turn rated as more attractive than the unattractive cat faces (*p* < 0.001). The effect of attractiveness for tiger faces was also significant, *F*(2, 69) = 272.86, *p* < 0.001, η*_*p*_*^2^ = 0.89, where the attractive tiger faces were more attractive than the average-looking tiger faces (*p* < 0.001), which was in turn more attractive than the unattractive tiger faces (*p* < 0.001).

##### Analysis of pleasure ratings

The main effect was significant for Attractiveness, *F*(2, 138) = 192.55, *p* < 0.001, η*_*p*_*^2^ = 0.74, but not for Face Type, *F*(1, 138) = 0.28, *p* = 0.60, η*_*p*_*^2^ = 0.002. These were qualified by a significant interaction, *F*(2, 138) = 36.02, *p* < 0.001, η*_*p*_*^2^ = 0.34. Analysis of simple effect showed that the attractive cat faces were rated as more pleasant than attractive tiger faces, *t*(45) = 6.95, *p* < 0.001, the unattractive cat faces were rated less pleasant than unattractive tiger faces, *t*(47) = −4.53, *p* < 0.001, but the difference between average-looking cat faces and tiger faces was not significant, *t*(46) = −0.99, *p* = 0.326. The effect of Attractiveness was significant for cat faces, *F*(2, 69) = 246.61, *p* < 0.001, η*_*p*_*^2^ = 0.88. Pairwise comparison (Bonferroni corrected) showed that the attractive cat faces was rated as more pleasant than the average-looking cat faces (*p* < 0.001), which was in turn rated as more pleasant than the unattractive cat faces (*p* < 0.001). The effect of Attractiveness was significant for tiger faces, *F*(2, 69) = 26.16, *p* < 0.001, η*_*p*_*^2^ = 0.43, where the attractive tiger faces was rated as more pleasant than the average-looking tiger faces (*p* = 0.004), which was in turn rated as more pleasant than the unattractive tiger faces (*p* = 0.001).

##### Analysis of arousal ratings

The main effect was significant for Attractiveness, *F*(2, 138) = 37.27, *p* < 0.001, η*_*p*_*^2^ = 0.35, and for Face Type *F*(1, 138) = 6.21, *p* = 0.014, η*_*p*_*^2^ = 0.04. These were qualified by a significant interaction, *F*(2, 138) = 6.07, *p* = 0.003, η*_*p*_*^2^ = 0.08. Analysis of simple effect showed that the arousal of attractive cat faces were higher than attractive tiger faces, *t*(45) = 4.20, *p* < 0.001. There were no significant difference between average-looking cat faces and tiger faces, or between unattractive cat faces and tiger faces, *t*s < 1.45, *p*s > 0.15. The effect of Attractiveness was significant for cat faces, *F*(2, 69) = 43.83, *p* < 0.001, η*_*p*_*^2^ = 0.56. Pairwise comparison (Bonferroni corrected) showed that the arousal of attractive cat faces was higher than average-looking cat faces, and unattractive cat faces, *p*s < 0.001, but the arousal between average-looking cat faces and unattractive cat faces was not significantly different, *p* = 0.507. The effect of Attractiveness was also significant for tiger faces, *F*(2, 69) = 7.93, *p* = 0.001, η*_*p*_*^2^ = 0.19, where the arousal of attractive tiger faces was higher than unattractive tiger faces (*p* = 0.001), and average-looking tiger faces (*p* = 0.05), but the arousal between average-looking tiger faces and unattractive tiger faces was not significantly different (*p* = 0.372).

##### Analysis of luminance and contrast

We calculated the luminance of each face by ImageJ^2^. We also programmed in Visual Basic and calculated the RMS contrast of each face (Wang et al., 2019). The mean luminance and contrast are given in Tables 4, 5. A two-way analysis of variance (ANOVA) was conducted on luminance, using Attractiveness and Face Type as independent variables. The main effect of Attractiveness was significant, *F*(2, 138) = 4.92, *p* = 0.009, η*_*p*_*^2^ = 0.07. The main effect of Face Type was not significant, *F*(1, 138) = 1.64, *p* = 0.203, η*_*p*_*^2^ = 0.01. The interaction was not significant, *F*(2, 138) = 0.57, *p* = 0.569, η*_*p*_*^2^ = 0.01.

**TABLE 4:** Mean luminance of cat and tiger faces in Experiments 1 and 2.

	Cat faces			Tiger faces		
	Attractive	Average-looking	Unattractive	Attractive	Average-looking	Unattractive
Experiment 1	133.12 (1.00)	132.62 (1.03)	132.67 (1.30)	132.95 (0.83)	132.19 (0.84)	132.66 (0.77)
Experiment 2	133.14 (0.95)	132.65 (0.49)	132.67 (1.60)	133.21 (0.82)	132.60 (0.88)	132.79 (0.92)

*Standard deviations are given in parentheses.*

**TABLE 5:** Mean RMS contrast of cat and tiger faces in Experiments 1 and 2.

	Cat faces			Tiger faces		
	Attractive	Average-looking	Unattractive	Attractive	Average-looking	Unattractive
Experiment 1	46.21 (4.23)	47.47 (4.34)	47.40 (6.08)	52.50 (2.51)	51.05 (2.23)	51.03 (2.60)
Experiment 2	46.00 (4.55)	46.10 (3.75)	48.82 (7.72)	51.70 (2.52)	50.53 (2.46)	51.48 (3.37)

*Standard deviations are given in parentheses.*

A two-way analysis of variance (ANOVA) was conducted on the RMS contrast, using Attractiveness and Face Type as independent variables. The main effect of Attractiveness was not significant, *F*(2, 138) = 0.02, *p* = 0.984, η*_*p*_*^2^ < 0.001. The main effect of Face Type was significant, *F*(1, 138) = 47.28, *p* < 0.001, η*_*p*_*^2^ = 0.26. The interaction was not significant, *F*(2, 138) = 1.86, *p* = 0.160, η*_*p*_*^2^ = 0.03.

Since there were differences in pleasure, arousal, luminance and contrast of faces among the experimental conditions, these factors were confounds. We took these factors as covariate variables in further analysis, in order to claim that the attractiveness of suppressed animal faces indeed played a role in gating suppression time.

##### Analysis of suppression time

In the b-CFS task, trials where the location was answered incorrectly or with suppression time more than three standard deviations above or below the mean of each condition in each participant were excluded. Overall, 5.6% of the trials were excluded from analyses. Pearson correlation was calculated between the mean ratings for faces and mean suppression time. There was a negative correlation between the two for cat faces (*r* = −0.28, *p* = 0.019), but not for tiger faces (*r* = −0.04, *p* = 0.759).

**FIGURE 3:**
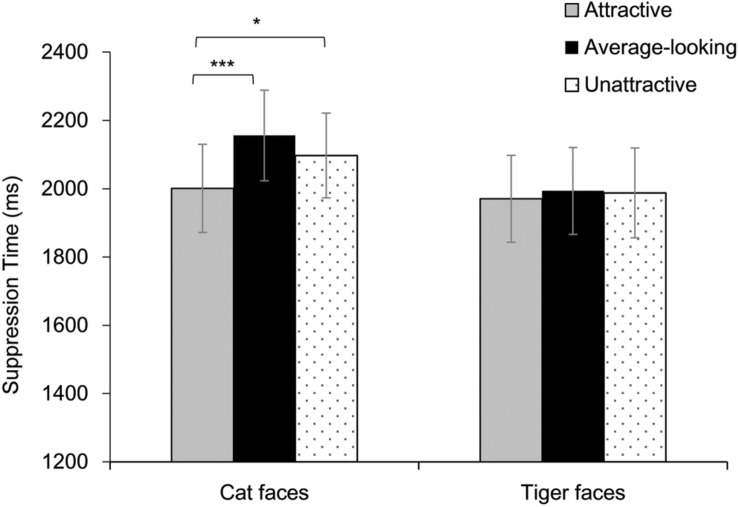
Suppression time for faces as a function of Attractiveness and Face Species in Experiment 1. Asterisks indicate a significant difference between conditions, *p < 0.05, ***p < 0.001. Error bars represent one standard error about the mean.

Results are shown in Figure 3. A two-way repeated-measures ANOVA showed a significant main effect of Attractiveness, *F*(2, 72) = 7.45, *p* = 0.001, η*_*p*_*^2^ = 0.17. The main effect of Face Type was not significant, *F*(1, 36) = 0.88, *p* = 0.355, η*_*p*_*^2^ = 0.02. These results were qualified by a significant interaction, *F*(2, 72) = 3.27, *p* = 0.044, η*_*p*_*^2^ = 0.08. Analysis of simple effect showed a significant main effect of Attractiveness for cat faces, *F*(2, 72) = 11.95, *p* < 0.001, η*_*p*_*^2^ = 0.25, but not for tiger faces, *F*(2, 72) = 0.16, *p* = 0.849, η*_*p*_*^2^ = 0.01. Further pairwise comparison (Bonferroni corrected) showed that attractive cat faces broke into awareness faster than average-looking cat faces and unattractive cat faces, *p*s ≤ 0.019. There was no difference between the average-looking and unattractive cat faces, *p* = 0.189.

Besides, one analysis of covariance (ANCOVA) was conducted on mean suppression time of each face, taking Attractiveness and Face Type as independent variables. Arousal, pleasure, luminance and contrast were taken as covariates. The main effect was significant for Face Type, *F*(1, 134) = 7.89, *p* = 0.006, η*_*p*_*^2^ = 0.06, but not for Attractiveness, *F*(2, 134) = 2.29, *p* = 0.105, η*_*p*_*^2^ = 0.03. These were qualified by a significant interaction, *F*(2, 134) = 3.36, *p* = 0.038, η*_*p*_*^2^ = 0.05. Analysis of simple effect showed that the main effect of attractiveness for cat faces was significant, *F*(2, 69) = 5.20, *p* = 0.008, η*_*p*_*^2^ = 0.13. Pairwise comparison (Bonferroni corrected) showed that attractive cat faces broke into awareness faster than average-looking cat faces, *p* = 0.009. The other pairwise comparisons were not significant, *p*s > 0.06. The main effect of attractiveness for tiger faces was not significant, *F*(2, 69) = 0.18, *p* = 0.838, η*_*p*_*^2^ = 0.01. Moreover, suppression time of averaging-looking tiger faces was shorter than cat faces, *t*(46) = −4.19, *p* < 0.001. Suppression time of unattractive tiger faces was shorter than cat faces, *t*(47) = −3.04, *p* = 0.004. There was no difference between the attractive tiger and cat faces, *t*(45) = −1.16, *p* = 0.254. The contrast covariate was significant, *F*(1, 134) = 4.79, *p* = 0.03, η*_*p*_*^2^ = 0.03, suggesting that the contrast of faces influenced suppression time. The effects of other covariates were not significant, *F*s ≤ 3.58, *p*s > 0.06.

The results thus show that observers were only more sensitive to attractiveness of cat faces. A possible explanation that only cat faces produced an attractiveness effect is that the difference between attractive and unattractive cat faces was much greater than that between attractive and unattractive tiger faces. The mean rating results in Table 1 shows the difference between attractive and unattractive faces was 3.30 (5.33–2.03) for cat faces but 1.85 (4.49–2.64) for tiger faces. To test whether the larger difference in attractiveness ratings resulted in the significant effect for cat faces, we reduced the scale of the difference by removing the results of top-rated cat faces from an additional analysis, where the rating results from seven attractive cat faces (*M* = 4.77, *SD* = 0.16) and eight unattractive cat faces (*M* = 2.21, *SD* = 0.33) were compared with eight attractive tiger faces (*M* = 4.77, *SD* = 0.11), and nine unattractive tiger faces (*M* = 2.27, *SD* = 0.15). ANOVA of the rating data for these selected faces showed a main effect of Attractiveness, *F*(1, 28) = 1188.17, *p* < 0.001, η*_*p*_*^2^ = 0.98. There was no effect of Face Type, *F*(1, 28) = 0.14, *p* = 0.708, η*_*p*_*^2^ = 0.01, or interaction between the two variables, *F*(1, 28) = 0.16, *p* = 0.689, η*_*p*_*^2^ = 0.01. Pairwise comparison (Bonferroni corrected) showed that the attractive cat faces were rated as more attractive than the unattractive cat faces (*p* < 0.001). The attractive tiger faces were rated as more attractive than the unattractive tiger faces (*p* < 0.001). Two-way ANOVAs were also conducted on the pleasure ratings, arousal ratings, luminance and contrast respectively. Results showed the main effect of Attractiveness was significant for arousal ratings, *F*(1, 28) = 8.37, *p* = 0.007, η*_*p*_*^2^ = 0.23, and for pleasure ratings, *F*(1, 28) = 178.58, *p* < 0.001, η*_*p*_*^2^ = 0.86. The main effect of Face Type was significant for contrast, *F*(1, 28) = 17.49, *p* < 0.001, η*_*p*_*^2^ = 0.39. The other effects were not significant, *F*s < 1.43, *p*s > 0.24. Therefore, pleasure, arousal and contrast were taken as covariates in further analysis.

Based on this new face stimulus set with equated attractiveness range for cat and tiger faces, we conducted a two-way repeated-measures ANOVA for their effects on suppression time. The main effect of Attractiveness was significant, *F*(1, 36) = 10.17, *p* = 0.003, η*_*p*_*^2^ = 0.22, whereas the effect of Face Type was not, *F*(1, 36) = 1.52, *p* = 0.226, η*_*p*_*^2^ = 0.04. There was a significant interaction between the two factors, *F*(1, 36) = 7.51, *p* = 0.009, η*_*p*_*^2^ = 0.17. Attractive cat faces broke into awareness faster than unattractive cat faces, *F*(1, 36) = 12.66, *p* = 0.001, η*_*p*_*^2^ = 0.26, but there was no difference between attractive and unattractive tiger faces, *F*(1, 36) = 0.13, *p* = 0.72, η*_*p*_*^2^ = 0.004. The results from this additional analysis were consistent with the analysis conducted on the whole face set. Thus the different ranges between attractive and unattractive levels in cat and tiger face stimuli cannot explain why only attractiveness of cat faces but not tiger faces had an effect in this task.

In addition, one analysis of covariance (ANCOVA) was conducted on the mean suppression time of each face based on this new face stimulus set, with Attractiveness and Face Type as independent variables, while taking arousal ratings, pleasure ratings and contrast as covariates. The main effects of Face Type and Attractiveness were not significant, *F*s ≤ 1.77, *p*s > 0.19. The interaction between Attractiveness and Face Type was significant, *F*(1, 25) = 4.86, *p* = 0.037, η*_*p*_*^2^ = 0.16. Analysis of simple effect showed that the attractive cat faces broke into awareness faster than unattractive cat faces, *F*(1, 13) = 14.72, *p* = 0.002, η*_*p*_*^2^ = 0.53. There was no difference between attractive and unattractive tiger faces, *F*(1, 15) = 0.14, *p* = 0.715, η*_*p*_*^2^ = 0.01. Unattractive tiger faces broke into awareness faster than unattractive cat faces, *t*(15) = −4.80, *p* < 0.001. There was no difference between attractive cat faces and attractive tiger faces, *t*(13) = 0.55, *p* = 0.593. The covariates were not significant, *F*s ≤ 4.08, *p*s > 0.05. Thus, the arousal, pleasure and contrast did not influence suppression time of the new face set with equated attractiveness range for cat and tiger faces.

Results of this experiment revealed that attractive cat faces broke into visual awareness more quickly. This is consistent with the previous research using human faces (Hung et al., 2016; Nakamura and Kawabata, 2018). To test the robustness of prioritization of special stimuli in awareness, past research has often investigated preconscious processing in both b-CFS and BR paradigms (Zhou et al., 2010; Stein et al., 2017). Following this tradition, our Experiment 2 aimed to test whether the attractiveness effect found for the cat faces could also be demonstrated with the BR paradigm.

## Experiment 2

### Materials and Methods

Employing the BR paradigm (Anderson et al., 2011), this experiment explored whether attractive animal faces would dominate the observer’s percept longer relative to less attractive ones. The participant was presented with an animal face to one eye but a house to the other eye simultaneously. The task was to report as soon as a face, a house or a mixture of the two was perceived.

#### Participants

Forty-six participants took part in the experiment for a payment of 30 RMB. All participants reported normal or corrected-to normal vision and were naïve to the purpose of the study.

Four participants were excluded following the same exclusion criterion as in Stein et al. (2017). These participants frequently exhibited unusually long dominance duration of a single percept, often lasted for the whole 10-s trial. As a result, few valid trials were left (less than 20%) for these participants after a large numbers of such trials were excluded. The final sample for the BR task consisted of 42 participants (33 females, *M*_age_ = 20.8, *SD*_age_ = 2.01).

#### Apparatus and Stimuli

Stimuli and instructions were presented on a 19-in CRT screen (1024 × 768 pixels resolution, 85 Hz refresh rate) using E-prime Version 2. The background of screen was black. The mirrors of the stereoscope (provided by Beijing Fistar Technology Co., Ltd.) were adjusted for each observer to support stable binocular fusion. A 2.16° × 3.11° frame that extended beyond the outer border of the stimulus and fixation point was presented to facilitate stable fusion of the two images.

Because each trial in this paradigm was quite long (10 s), it was not practical to test all 288 face images used in Experiment 1. Instead, we chose 10 attractive, 10 average-looking, and 10 unattractive faces from each of the two face types based on the ratings used in Experiment 1. The second row of Table 1 shows attractiveness ratings for the cat and tiger faces used in this experiment. The mean ratings of pleasure and arousal for cat and tiger faces were shown in the second row of Tables 2, 3 respectively. The mean luminance and RMS contrast of cat and tiger faces were shown in the second row of Tables 4, 5 respectively. The 60 house photos were adopted from Anderson et al. (2011). They were gray-scale and were scaled to the same size, luminance and contrast as the faces using Adobe Photoshop CS. Each face was paired with one house.

#### Design

We employed a within-subject design. The independent variables were Face Type (cat vs. tiger) and Attractiveness (attractive, average-looking, unattractive). The dependent variable was the mean dominance durations.

#### Procedure

**FIGURE 4:**
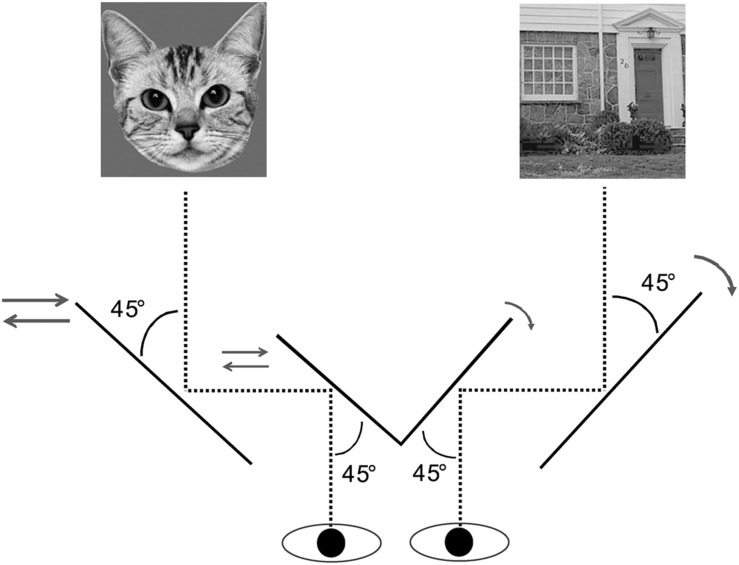
Illustration of mirror stereoscope setup for the BR paradigm in Experiment 2.

Participants viewed the screen dichoptically through a mirror stereoscope with their head fixed on a chin rest at a distance of 66 cm. At the beginning of each trial, a red 0.09° × 0.09° fixation dot was presented centrally for 1 s. Subsequently, a face-house pair was displayed for 10 s. As illustrated in Figure 4, the face was presented to one eye, whereas the house was presented to the other. Consecutive trials were separated by a blank screen with the introduction “press any key to continue.” Participants were asked to press the left arrow when they saw a face, the right arrow key when they saw a house, and the down arrow key when they saw a mixture of the two. They were instructed to indicate their percept continuously by pressing one of the three keys throughout a trial.

The cat faces and tiger faces were presented in separate blocks. Each block had 60 trials, where the 30 cat or tiger faces were shown twice, once to the left eye and once to the right eye. Each cat/tiger face was paired with one of the 60 houses. The order of these trials within each block was random, whereas the order of the two blocks was counterbalanced across participants. At the beginning of each block, participants were given 12 practice trials with example stimuli that were not used in the subsequent experimental trials. Participants were given a break between the blocks.

#### Results and Discussion

As Experiment 1, ANOVAs were conducted on the mean attractiveness, pleasure and arousal ratings for cat and tiger faces separately, with Attractiveness and Face Type as the fixed factors.

##### Analysis of attractiveness

Significant main effects were found for Attractiveness, *F*(2, 54) = 1050.75, *p* < 0.001, η*_*p*_*^2^ = 0.98, and Face Type, *F*(1, 54) = 15.81, *p* < 0.001, η*_*p*_*^2^ = 0.23. There was also a significant interaction between these, *F*(2, 54) = 67.52, *p* < 0.001, η*_*p*_*^2^ = 0.71. Analysis of simple effect showed that the attractive cat faces were more attractive than attractive tiger faces, *F*(1, 18) = 97.46, *p* < 0.001, η*_*p*_*^2^ = 0.84, the average-looking cat faces were also more attractive than average-looking tiger faces, *F*(1, 18) = 8.40, *p* = 0.01, η*_*p*_*^2^ = 0.32, but the unattractive cat faces were more unattractive than unattractive tiger faces, *F*(1, 18) = 44.83, *p* < 0.001, η*_*p*_*^2^ = 0.71. ANOVAs on the mean ratings were also conducted separately for cat and tiger faces. The effect of attractiveness was significant for cat faces, *F*(2, 27) = 662.29, *p* < 0.001, η*_*p*_*^2^ = 0.98. Pairwise comparison (Bonferroni corrected) showed higher attractive ratings for attractive than for average-looking cat faces (*p* < 0.001), which were in turn rated more attractive than unattractive cat faces (*p* < 0.001). The effect of attractiveness was also significant for tiger faces, *F*(2, 27) = 338.70, *p* < 0.001, η*_*p*_*^2^ = 0.97. Ratings for attractive tiger faces were higher than average-looking tiger faces (*p* < 0.001), which were in turn rated higher than unattractive tiger faces (*p* < 0.001).

##### Analysis of pleasure ratings

Significant main effect was found for Attractiveness, *F*(2, 54) = 188.83, *p* < 0.001, η*_*p*_*^2^ = 0.88, but not for Face Type, *F*(1, 54) = 0.86, *p* = 0.358, η*_*p*_*^2^ = 0.02. There was a significant interaction between Attractiveness and Face Type, *F*(2, 54) = 14.42, *p* < 0.001, η*_*p*_*^2^ = 0.35. Analysis of simple effect showed that the attractive cat faces were rated as more pleasant than attractive tiger faces, *t*(18) = 4.70, *p* < 0.001. The unattractive tiger faces were rated as more pleasant than unattractive cat faces, *t*(18) = 2.58, *p* = 0.019. The pleasure of average-looking cat faces was not significantly different from average-looking tiger faces, *t*(18) = −0.45, *p* = 0.661. The effect of Attractiveness was significant for cat faces, *F*(2, 27) = 187.22, *p* < 0.001, η*_*p*_*^2^ = 0.93. Pairwise comparison (Bonferroni corrected) showed that the attractive cat faces were rated as more pleasant than for average-looking cat faces (*p* < 0.001), which were in turn rated as more pleasant than unattractive cat faces (*p* < 0.001). The effect of Attractiveness was also significant for tiger faces, *F*(2, 27) = 43.21, *p* < 0.001, η*_*p*_*^2^ = 0.76. Pairwise comparison (Bonferroni corrected) showed attractive tiger faces were rated as more pleasant than for average-looking tiger faces (*p* = 0.007), which were in turn rated as more pleasant than unattractive tiger faces (*p* < 0.001).

##### Analysis of arousal ratings

Significant main effect was found for Attractiveness, *F*(2, 54) = 28.09, *p* < 0.001, η*_*p*_*^2^ = 0.51, but not for Face Type, *F*(1, 54) = 0.80, *p* = 0.374, η*_*p*_*^2^ = 0.02. There was a significant interaction between Attractiveness and Face Type, *F*(2, 54) = 4.76, *p* = 0.013, η*_*p*_*^2^ = 0.15. Analysis of simple effect showed that the arousal ratings of attractive cat faces was higher than attractive tiger faces, *t*(18) = 4.14, *p* = 0.001. Neither the difference in arousal ratings between average-looking cat faces and tiger faces nor the difference in arousal ratings between unattractive cat faces and tiger faces was significant, *t*s < 1.34, *p*s > 0.19. The effect of Attractiveness was significant for cat faces, *F*(2, 27) = 47.73, *p* < 0.001, η*_*p*_*^2^ = 0.78. Pairwise comparison (Bonferroni corrected) showed arousal ratings of attractive cat faces were higher than average-looking cat faces and unattractive cat faces (*p*s < 0.001). But the difference between average-looking cat faces and unattractive cat faces was not significant, *p* > 0.99. The effect of Attractiveness was significant for tiger faces, *F*(2, 27) = 3.58, *p* = 0.042, η*_*p*_*^2^ = 0.21. But there is no significant effect in pairwise comparisons (*p*s > 0.05).

##### Analysis of luminance and contrast

As in Experiment 1, ANOVAs were conducted separately on the luminance and RMS contrast for the faces with Attractiveness and Face Type as the fixed factors. We only found the contrast of tiger faces was higher than cat faces, *F*(1, 54) = 13.91, *p* < 0.001, η*_*p*_*^2^ = 0.21. Other effects were not significant, *F*s ≤ 1.70, *p*s > 0.19.

Since there were differences in pleasure, arousal and contrast of faces among the experimental conditions, we took these factors as covariate variables in further analysis, in order to claim that the attractiveness of suppressed animal faces indeed played a role in gating dominance durations.

##### Analysis of dominance durations

Mean dominance durations were calculated for attractive, average-looking, unattractive cat and tiger faces. Following Anderson et al. (2011) and Stein et al. (2017), percepts at the end of the 10-s trials were excluded as they were artificially shortened. Therefore, the trials with only one percept dominating the whole 10-s were excluded. Moreover, the percepts whose durations were less than 100 ms were also excluded (0.2% of the percepts). For the effective percepts, the percentage of mixed percepts was 34.9%. The percentage of face percepts was 37.3%. The percentage of house percepts was 27.8%.

**FIGURE 5:**
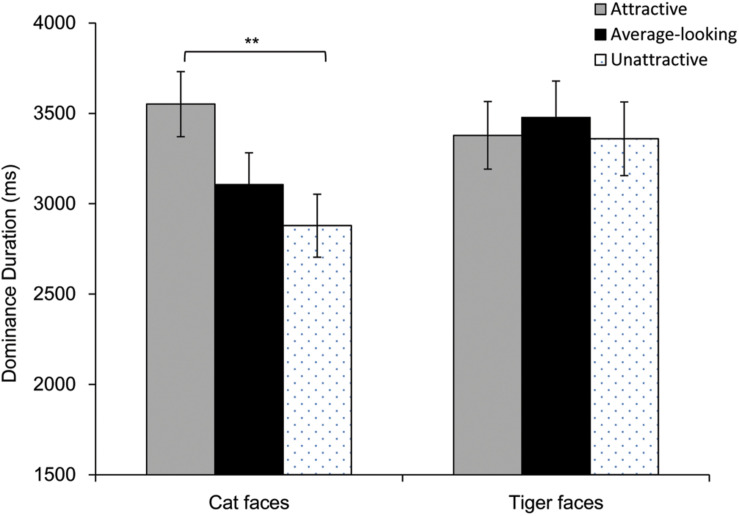
Dominance duration as a function of Attractiveness and Face Type in Experiment 2. Error bars represent one standard error about the mean. Asterisks indicate a significant difference between conditions, **p < 0.01.

Results of dominance durations are shown in Figure 5. Firstly, we calculated Pearson correlation between the mean ratings and mean dominance durations. There was a highly positive correlation for cat faces (*r* = 0.46, *p* = 0.011), whereas there was not a correlation for tiger faces (*r* = 0.17, *p* = 0.358). Then a repeated-measures ANOVA was performed on mean dominance duration. Greenhouse-Geisser correction was adopted when the assumption of sphericity was violated. The main effect was significant for Attractiveness, *F*(1.72, 70.65) = 3.49, *p* = 0.042, η*_*p*_*^2^ = 0.08, but not for Face Type, *F*(1, 41) = 3.94, *p* = 0.054, η*_*p*_*^2^ = 0.09. There was however a significant interaction between these, *F*(1.66, 67.96) = 4.01, *p* = 0.029, η*_*p*_*^2^ = 0.09. Analysis of simple effect showed that main effect of attractiveness was significant for cat faces, *F*(2, 82) = 6.73, *p* = 0.002, but not for tiger faces, *F*(2, 82) = 0.27, *p* = 0.767. Pairwise comparison (Bonferroni corrected) showed that attractive cat faces dominated longer than unattractive cat faces, *p* = 0.007, although dominance duration for the average-looking cat faces was not different from that for attractive or unattractive cat faces, *p*s > 0.09.

As Experiment 1, one analysis of covariance (ANCOVA) was conducted on mean dominance duration of each face, with Attractiveness and Face Type as independent variables, while taking arousal, pleasure and contrast were covariates. The main effect of Attractiveness was significant, *F*(2, 51) = 3.36, *p* = 0.043, η*_*p*_*^2^ = 0.12. Pairwise comparison (Bonferroni corrected) showed that attractive faces dominated longer than unattractive faces, *p* = 0.037, but dominance duration for the average-looking faces was not different from attractive or unattractive faces, *p*s > 0.103. However, the main effect of Face Type, and the interaction between Attractiveness and Face Type were not significant, *F*s ≤ 3.08, *p*s > 0.05. The pleasure covariate, arousal covariate and the contrast covariate were not significant, *F*s < 1.84, *p*s > 0.18. These covariates did not influence dominance duration.

Consistent with Experiment 1, repeated-measures ANOVA based on mean dominance duration of each participant showed that the effect of attractiveness was again found for cat faces, but not for tiger faces. In addition, the ANCOVA based on mean dominance duration of each face showed that both attractive cat and tiger faces dominated longer than unattractive faces. However, unlike Experiment 1, we were unable to rule out the explanation that the effect for cat faces could be due to the larger difference between the levels of attractive and unattractive cat faces related to the range of attractive level in the tiger faces. In Experiment 1, we were able to use a balanced range of attractiveness for the two types of faces by removing the results of faces that contributed to the imbalance. However, because there were only 10 faces for level of attractiveness in this experiment and because the mean rating of every attractive cat faces was higher than the mean rating of every attractive tiger face, we were unable to do the same analysis. Nevertheless, from the results in Experiment 1, we learned that this analysis is not likely to change the null finding for the tiger faces. It is also less important to show here again that even a reduced range of attractiveness in the stimuli could still produce an attractiveness effect for cat faces, because our key purpose was simply to determine whether human observers could demonstrate the same attractiveness effect with animal faces that were previously only shown in perception of human faces.

## General Discussion

The advantage of attractive faces in a human observer’s preconscious processing has only been studied using conspecific face images. Attractive human faces are detected more quickly and get access to consciousness in the b-CFS paradigm (Nakamura and Kawabata, 2014; Hung et al., 2016), and persisted longer in the BR task (Mo et al., 2016). However, it is unknown whether this advantage is specific to human faces. The current study investigated whether the same attractiveness effects could be found for faces of non-human animals. In the b-CFS task (Experiment 1), attractive cat faces reached visual awareness more quickly than unattractive cat faces. In the BR task (Experiment 2), attractive cat faces dominated visual awareness longer as compared to less attractive cat faces. However, attractiveness of tiger faces did not produce as much effects as that of cat faces.

Given the cat face attractiveness effects in this study, it is clear that the processing advantage for attractive human faces found in the literature cannot be solely attributed to the adaptive function of mate choice. Our results show that facial attractiveness may be pursued for its own sake rather than for the purpose of natural selection. This finding may support the hypothesis that preference for attractive faces is a by-product of how brains process information rather than an adaptive mechanism for mate choice (see Rhodes, 2006, for a review). However, there is also a possibility of a mixed account where attractiveness processing on human-like stimuli originates from mate choice while attractiveness processing on non-human-like stimuli is simply by-products of the previous kind. Thus, the findings of the current study can be neutral to such a debate.

The perceptual advantage for attractive cat faces in the present study is consistent with prior research that demonstrated infants’ preferences for attractive cat faces (Quinn et al., 2008). However, unlike Quinn et al. (2008) who found the attractiveness effects for both cat and tiger faces, the experiments in this study only found an advantage of processing attractive cat faces in b-CFS task, while found an advantage of processing attractive cat and tiger faces in BR task (ANCOVA results). This creates an apparent conflict between the data of infants and adults. However, it should be noted that our adult participants could also distinguish attractiveness of tiger faces in the rating task. Their rating results showed good inter-rater reliability. The three levels of attractiveness were significantly different from each other. In this respect, the tiger rating results were in fact consistent with the results from the infant study. The real difference between the results of the cat and tiger faces in our study is likely due to the degree of sensitivity to the attractiveness of the two types of faces. From the rating results alone, it can be seen that participants produced a greater range of attractiveness scores for cat faces relative to their scores for tiger faces. There is also a trend that inter-rater reliability (Kendall’s W) was larger for cats (0.56) than for tigers (0.23). This may suggest that our participants were more sensitive and more able to discriminate facial attractiveness among cats. This would be consistent with the theory of perceptual narrowing (Nelson, 2001), because people’s sensitivity to attractiveness may be more finely tuned to cats due to their more frequent contact with them. Indeed, familiarity is considered as one of the factors that affect preconscious processing of faces (Axelrod et al., 2015).

Consistent with this interpretation, both the b-CFS and the BR tasks require sensitivity to attractiveness discrimination. The less attractiveness effect for the tiger faces in these tasks may due to lower sensitivity or ability to discriminate attractiveness in tiger faces. We tried to equate the attractiveness levels for the two types of faces in Experiment 1. The results based on the matched attractiveness levels from selected cat and tiger faces replicated the results based on all faces. That is, the effect of attractiveness of cat faces persisted, while the effect of tiger faces remained absent. It is important to note that matching the ranges of attractiveness for the two face types only reduces the range of attractiveness for the cat faces. It does not increase the range of attractiveness for the tiger faces. Hence it is not surprising that the analysis based on matched attractive range did not show effect of attractiveness for tiger faces. Rather, it is interesting that cat faces still showed the effect of attractiveness after their range of attractiveness was compressed. Along with the rating results, this may be a further demonstration that participants were particularly sensitive to cat facial attractiveness.

There are some limitations in the present study. First, since the cat faces and tiger faces were collected from internet, it is difficult to control the low-level properties such as gaze direction, eye position, size of the eyes, symmetry of the faces, head angles to the observer and the identity recognizability of the cat faces and tiger faces. These differences in low-level visual features of cat and tiger faces could affect the results (Stein et al., 2011, 2018; Gray et al., 2013; Nakamura and Kawabata, 2014; Moors et al., 2017, 2019). In order to show that it is attractiveness but not other potential confounds drove the difference in perception, future research should use a control experiment to show that the attractiveness effect disappears when the target images are inverted (e.g., Gayet et al., 2014). This control experiment preserves most of the local perceptual components while disrupts face-level attractiveness processing. Secondly, in Experiment 2, the participants did not rate the attractiveness of the stimuli. We used attractiveness ratings of Experiment 1 to select stimuli. There may be potential individual differences in rating the attractiveness between the two experiments. Thirdly, there were other confounds of the stimuli in the present research: pleasure, arousal. Although the luminance and contrast of the stimuli were normalized using Adobe Photoshop CS (Anderson et al., 2011), this software did not calculate the luminance and contrast of each face. Further calculation showed that these values varied across faces. However, the analysis of covariance (ANCOVA) for suppression time and dominance duration of each face still showed effect of attractiveness after taking these confounds as covariates. Future research should use larger face database and select stimuli which are only different in attractiveness.

In sum, the main purpose of this study was to find out whether human observers would show similar sensitivity to attractiveness in animal faces as they usually do to human faces. Our results show that this was at least the case with cat faces. The present study provides the first evidence that effects of cat facial attractiveness can be demonstrated through the b-CFS and BR paradigms. The results suggest that human preference for attractive faces is not limited to the purpose for potential mates.

## Data Availability Statement

The separate data analysis based on different genders for this study are included in the article/Supplementary Material, further inquiries can be directed to the corresponding authors.

## Ethics Statement

The studies involving human participants were reviewed and approved by the Institutional Review Board of the Liaoning Normal University, China. The participants provided their written informed consent to participate in this study.

## Author Contributions

JS, WC, and CL designed the experiment and revised the manuscript. ZL, HY, CW, and LZ prepared the materials and performed the experiment. JS, ZL, CW, and LZ analyzed the data. ZL and HY wrote the manuscript. All authors contributed to the article and approved the submitted version.

## Conflict of Interest

The authors declare that the research was conducted in the absence of any commercial or financial relationships that could be construed as a potential conflict of interest.

## Footnotes

1 https://www.wjx.cn/

2 https://imagej.nih.gov/ij/download.html
