# Supplementary material for: Perceptual Advantage of Animal Facial Attractiveness: Evidence From b-CFS and Binocular Rivalry
Source: Front Psychol. 2020 Jul 10;11:1670. doi: 10.3389/fpsyg.2020.01670 (PMC7367084; doi:10.3389/fpsyg.2020.01670)
Supplement: Supplementary file 1 [file Data_Sheet_1.PDF]

Separate data analysis from the two genders

Experiment 1

Analysis of male participants based on the whole faces: A two-way repeated-measures ANOVA showed a significant main effect of Attractiveness, $F(2,6) = 26.57$, $p = 0.001$, $\eta_p^2 = 0.90$. The main effect of Face Type was not significant, $F(1,3) = 0.006$, $p = 0.942$, $\eta_p^2 = 0.002$. The interaction between Attractiveness and Face Type was not significant, $F(2,6) = 2.94$, $p = 0.129$, $\eta_p^2 = 0.50$. Pairwise comparison (Bonferroni corrected) showed that attractive faces broke into awareness faster than average-looking cat faces, p=0.025. The other pairwise comparisons were not significant, ps>0.05.

Besides, one analysis of covariance (ANCOVA) was conducted on mean suppression time of each face, taking Attractiveness and Face Type as independent variables. Arousal, pleasure, luminance and contrast were taken as covariates. The main effect of Attractiveness was significant, $F(2,134) = 4.23$, p =0.017, $\eta_p^2 = 0.06$. The main effect of Face Type was not significant, $F(1,134) = 0.45$, $p = 0.502$, $\eta_p^2 = 0.003$. The interaction was not significant, $F(2,134) = 2.16$, $p = 0.119$, $\eta_p^2 = 0.03$. Pairwise comparison (Bonferroni corrected) showed that attractive faces broke into awareness faster than average-looking cat faces, p=0.018. The other pairwise comparisons were not significant, ps>0.28. The effects of all covariates were not significant, Fs < 1.11, ps> 0.29.

Analysis of female participants based on the whole faces: A two-way repeated-measures ANOVA showed a significant main effect of Attractiveness, $F(2,64) = 4.20$, $p = 0.019$, $\eta_p^2 = 0.12$. The main effect of Face Type was not significant, $F(1,32) = 1.09$, $p = 0.303$, $\eta_p^2 = 0.03$. The interaction between Attractiveness and Face Type was not significant, $F(2,64) = 2.44$, $p = 0.096$, $\eta_p^2 = 0.07$. Pairwise comparison (Bonferroni corrected) showed that attractive faces broke into awareness faster than average-looking cat faces, p=0.025. The other pairwise comparisons were not significant, ps>0.14.

Besides, one analysis of covariance (ANCOVA) was conducted on mean suppression time of each face, taking Attractiveness and Face Type as independent variables. Arousal, pleasure, luminance and contrast were taken as covariates. The main effect of Face Type was significant, $F(1,134) = 10.97$, p =0.001, $\eta_p^2 = 0.08$. The main effect of Attractiveness was not significant, $F(2,134) = 1.18$, $p = 0.311$, $\eta_p^2 = 0.02$. The interaction was not significant, $F(2,134) = 2.70$, $p = 0.071$, $\eta_p^2 = 0.04$. The contrast covariate was significant, $F(1,134) = 5.09$, $p = 0.026$, $\eta_p^2 = 0.04$, suggesting that the contrast of faces influenced suppression time. The effects of other covariates were not significant, Fs < 3.26, ps> 0.07.

Analysis of male participants based on selected faces: Based on this new face stimulus set with equated attractiveness range for cat and tiger faces, we conducted a two-way repeated-measures ANOVA on suppression time. The main effect of Attractiveness was not significant, $F(1,3)$ =9.10, $p = 0.057$, $\eta_p^2 = 0.75$. The main effect of Face Type was not significant, $F(1,3) = 0.04$, $p = 0.849$, $\eta_p^2 = 0.01$. The interaction between the two factors was not significant, $F(1,3) = 1.63$, $p = 0.291$, $\eta_p^2 = 0.35$.

In addition, one analysis of covariance (ANCOVA) was conducted on the mean suppression time of each face based on this new face stimulus set, with Attractiveness and Face Type as independent variables, while taking arousal ratings, pleasure ratings and contrast as covariates. The main effect of Attractiveness was significant, $F(1,25)$=4.36, $p = 0.047$, $\eta_p^2 = 0.15$, but the effect of Face Type was not significant, $F(1,25)$=3.48, $p = 0.074$, $\eta_p^2 = 0.12$. The interaction

between Attractiveness and Face Type was significant, $F(1,25) = 4.41$, $p = 0.046$, $\eta_p^2 = 0.15$. Analysis of simple effect showed that the attractive cat faces broke into awareness faster than unattractive cat faces, $t(13) = -3.20$, $p = 0.007$. There was no difference between attractive and unattractive tiger faces, $t(15) = -0.22$, $p = 0.825$. Attractive cat faces broke into awareness faster than attractive tiger faces, $t(13)= -2.35$, $p = 0.035$. There was no difference between unattractive cat faces and unattractive tiger faces, $t(15)= 0.73$, $p = 0.475$. The covariates were not significant, Fs < 3.56, ps > 0.07. Thus, the arousal, pleasure and contrast would not influence suppression time of the new face set with equated attractiveness range for cat and tiger faces.

Analysis of female participants based on selected faces: Based on this new face stimulus set with equated attractiveness range for cat and tiger faces, we conducted a two-way repeated-measures ANOVA on suppression time. The main effect of Attractiveness was significant, $F(1,32) =6.88$, $p = 0.013$, $\eta_p^2 = 0.18$. The main effect of Face Type was not significant, $F(1,32) = 1.91$, $p = 0.177$, $\eta_p^2 = 0.06$. The interaction between the two factors was significant, $F(1,32) = 5.76$, $p = 0.022$, $\eta_p^2 = 0.15$. Analysis of simple effect showed that the attractive cat faces broke into awareness faster than unattractive cat faces, $F(1,32) =8.81$, $p = 0.006$, $\eta_p^2 = 0.22$. There was no difference between attractive and unattractive tiger faces, $F(1,32) =0.07$, $p = 0.798$, $\eta_p^2 = 0.002$.

In addition, one analysis of covariance (ANCOVA) was conducted on the mean suppression time of each face based on this new face stimulus set, with Attractiveness and Face Type as independent variables, while taking arousal ratings, pleasure ratings and contrast as covariates. The main effects of Attractiveness and Face Type were not significant, Fs < 3.12, ps > 0.09. The interaction between Attractiveness and Face Type was also not significant, $F(1,25) =2.79$, $p = 0.107$, $\eta_p^2 = 0.10$. The covariates were not significant, Fs <2.31, ps > 0.14.

Experiment 2

Analysis for male participants: A repeated-measures ANOVA was performed on mean dominance duration. The main effects of Attractiveness and Face Type were not significant, Fs ≤ 1.47, ps > 0.26. The interaction between Attractiveness and Face Type was also not significant, $F(1.25,10.02) =0.26$, $p = 0.674$, $\eta_p^2 = 0.03$.

One analysis of covariance (ANCOVA) was conducted on mean dominance duration of each face, with Attractiveness and Face Type as independent variables, while taking arousal, pleasure and contrast as covariates. The main effects of Attractiveness and Face Type were not significant, Fs < 1.44, ps > 0.24. The interaction was also not significant, $F(2,51) =0.05$, $p = 0.951$, $\eta_p^2 = 0.002$. The arousal covariate was significant, $F(1,51) = 14.45$, $p < 0.001$, $\eta_p^2 = 0.22$, suggesting that the arousal of faces influenced dominance duration. The effects of other covariates were not significant, Fs < 0.48, ps> 0.49.

Analysis for female participants: A repeated-measures ANOVA was performed on mean dominance duration. The main effect of Attractiveness was significant, $F(2, 62) = 7.21$, $p = 0.002$, $\eta_p^2 = 0.19$. The main effect of Face Type was not significant, $F(1, 31) = 1.75$, $p = 0.196$, $\eta_p^2 = 0.05$. There was a significant interaction between these, $F(2, 62) = 7.83$, $p = 0.001$, $\eta_p^2 = 0.20$. Analysis of simple effect showed that main effect of attractiveness was significant for cat faces, $F(2, 62) = 12.58$, $p < 0.001$, $\eta_p^2 = 0.29$. Pairwise comparison (Bonferroni corrected) showed that attractive faces broke into awareness faster than average-looking cat faces and unattractive cat faces, ps<0.01. But there was no difference between average-looking cat faces and unattractive cat faces, p=0.411.

One analysis of covariance (ANCOVA) was conducted on mean dominance duration of each face, with Attractiveness and Face Type as independent variables, while taking arousal, pleasure and contrast as covariates. The main effect of Attractiveness was significant, $F(2,51) = 4.26$, $p = 0.019$, $\eta_p^2 = 0.14$. The main effect of Face Type was not significant, $F(1,51) < 0.001$, $p = 0.986$, $\eta_p^2 < 0.001$. The interaction between Attractiveness and Face Type was significant, $F(2,51) = 3.68$, $p = 0.032$, $\eta_p^2 = 0.13$. Analysis of simple effect showed that the main effect of attractiveness for cat faces was significant, $F(2,27) = 6.92$, $p = 0.004$, $\eta_p^2 = 0.34$. Pairwise comparison (Bonferroni corrected) showed that attractive cat faces dominated longer than unattractive cat faces, $p = 0.003$. The other pairwise comparisons were not significant, ps>0.06. The main effect of attractiveness for tiger faces was not significant, $F(2,27) = 0.16$, $p = 0.329$, $\eta_p^2 = 0.08$. The pleasure covariate, arousal covariate and the contrast covariate were not significant, Fs < 3.10, ps >0.08.
